# The Role of Women Authors and Editors in the *Balkan Medical Journal* in the Last Decade

**DOI:** 10.4274/balkanmedj.galenos.2019.2019.4.002

**Published:** 2019-07-11

**Authors:** İrem İNANÇ, Mustafa İNAN

**Affiliations:** 1 Department of Pediatric Surgery, Trakya University School of Medicine, Edirne, Turkey

When we examine the history of science, we can witness women with significant achievements being underappreciated by scientific societies across the world. Very few women, such as the recipients of the Nobel Science Awards, including Marie Curie, Dorothy Hodgkin, Maria Goeppert-Mayer, Donna Strickland, Gerty Theresa Cori, and Françoise Barre-Sinoussi, have achieved significant success through arduous struggles. Taking a closer look at the challenges that female scientists face clearly demonstrates that they are largely marginalized by their male colleagues. However, it is our belief that female scholars and authors have been taking a more active role in the scientific world in recent years. This editorial aims to examine the reflections of this case on the *Balkan Medical Journal* as an international medical journal.

The archives for the last decade of the Journal were scanned, featuring 1048 articles, including the first issue of 2008 and the last issue of 2018, to calculate the ratio of female/male authors. In addition, the ratio of female/male authors with first and last names in publications from non-Turkish Balkan countries (NTBC) as well as the female/male ratio of the editorial board members of the *Balkan Medical Journal* was examined. The gender information of all authors and editorial board members was accessible. Data were processed in an Excel file and evaluated using parameters defining male and female sexes.

When the gender ratio of first authors in all articles published in the *Balkan Medical Journal* was examined, the ratio of female authors to male authors was 0.47 in 2008, which increased to 0.57 in 2018. The gender distribution of last authors was 0.37 in 2008 and 0.29 in 2018, although a general tendency of increase was identified (Figure 1).

The first study from NTBC was published in 2010 with the female/male ratio of first authors for a total of 68 papers being 0.5, which increased to 1 by the end of 2018. The gender ratio for last authors of papers in this group was also similar (Figure 2).

In 49 issues published during the same period, it was observed that the first female member joined the editorial board in 2012 and the female/male ratio reached 0.38 in 2018 (Figure 3).

In the publications of medicine, it has been assumed that the first author makes the most contribution, whereas the last author typically functions as a mentor (1). Based on this assumption, an evaluation of all issues of the Journal in the last decade revealed no significant increase in the number of female last authors. However, the figure for female authors clearly indicated that female authors gained prominence. A study of six medical journals with high impact factor *(Annals of Internal Medicine, Archives of Internal Medicine, The BMJ, JAMA, The Lancet, and The New England Journal of Medicine)* comparing the author genders in the papers for the past 20 years found that the ratio of female authors was 0.27 in 1994 and increased to only 0.37 in 2014 (2). This figure is significant as it reveals the inadequate presence of female authors in prominent journals of medicine in the literature published in English language.

Similar to the data for the *Balkan Medical Journal*, the literature indicates a significant increase in the ratio of female first authors in recent years, with no distinct change in the ratio of female last authors (3,4). These data are significant as they indicate that the male dominance in the position of final author has remained virtually unchanged and women could not be adequately present in positions of leadership or mentoring.

Examining the NTBC publications for first and last authors showed no significant change in the ratio of female authors. However, the charts indicate that the ratio of female authors in NTBC-origin papers is higher than the ratio of female authors in papers from other countries. This may be interpreted as a consequence of the educational and scientific policies implemented in NTBC after World War II.

An increase in the number of female editors in editorial boards in recent years is also evident. Although the *Balkan Medical Journal* had no female editors in the first few years included in the study, the female/male ratio in 2018 soared to 0.38, which highlights an important change. This may imply that women are a predominant driving force in increasing the impact factor of the *Balkan Medical Journal*. On the contrary, it is noteworthy that no woman has ever served as a chief editor (5).

In conclusion, the increase in the number of female authors in the *Balkan Medical Journal* is significant, although the levels in NTBC have not yet been achieved, and a higher increase in the coming decade will bring us closer to equally representing both genders in the future.

It is also evident that the social perception of gender and equality is changing very slowly in the academic field. Although significant progress has been made in gender equality, it is a fact that women cannot adequately attain their rightful place in the academic world.

## Figures and Tables

**Figure 1:**
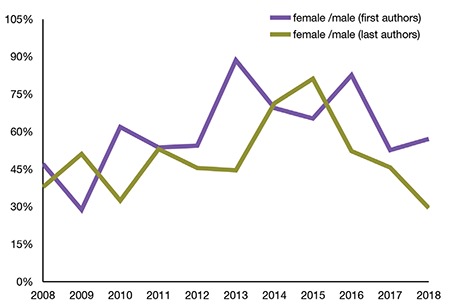
Female/male gender ratio of first and last authors.

**Figure 2:**
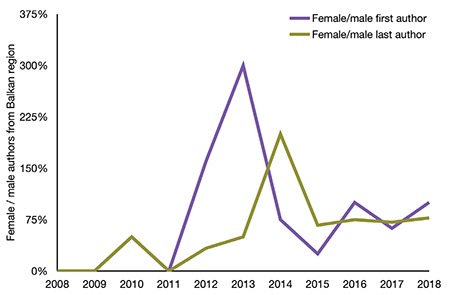
Female/male gender ratio of first and last authors from non-Turkish Balkan countries.

**Figure 3:**
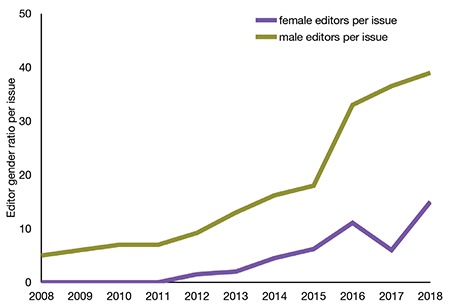
Female/male gender ratio of the editorial board.
